# Supplementary material for: Comparative multiomics analysis of cell physiological state after culture in a basket bioreactor
Source: Sci Rep. 2022 Nov 23;12:20161. doi: 10.1038/s41598-022-24687-4 (PMC9686226; doi:10.1038/s41598-022-24687-4)
Supplement: Supplementary file 1 — Supplementary Information 1. [file 41598_2022_24687_MOESM1_ESM.zip › raw data/Transcriptomic raw data/4.Quant/3.Distribution/density.pdf]

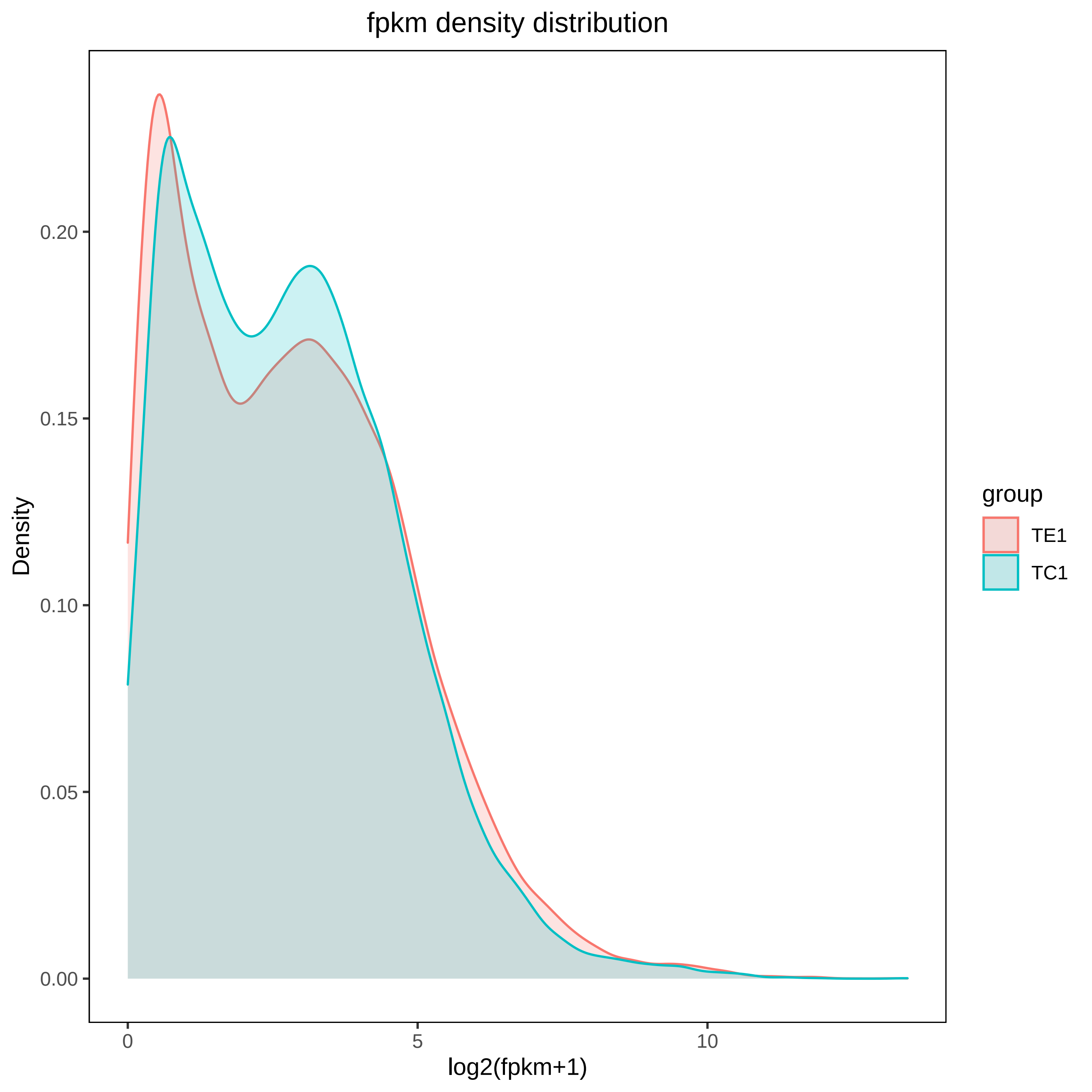

fpkm density distribution
0.00
0.05
0.10
0.15
0.20
Density
0
5
10
log2(fpkm+1)
group
TE1
TC1
